# Repetitive negative thinking mediates the relationship between addictive Facebook use and suicide-related outcomes: A longitudinal study

**DOI:** 10.1007/s12144-021-02025-7

**Published:** 2021-06-28

**Authors:** Julia Brailovskaia, Jürgen Margraf, Tobias Teismann

**Affiliations:** Mental Health Research and Treatment Center, Department of Clinical Psychology and Psychotherapy, Ruhr-Universität Bochum, Massenbergstraße 9-13, 44787 Bochum, Germany

**Keywords:** Addictive Facebook use, Repetitive negative thinking, Suicide ideation, Suicide attempts, Mediation model

## Abstract

Repetitive negative thinking (RNT) and addictive Facebook use have been shown to be associated with suicide ideation and suicidal behavior. The present longitudinal study aimed to investigate whether the association between addictive Facebook use and suicide-related outcomes is mediated by RNT. Data of 191 German Facebook users (*M*_*age*_ = 26.17, *SD*_*age*_ = 6.63) were assessed at two measurement time points over a one-year period (first measurement = T1, second measurement = T2) via online surveys. The significant positive association between addictive Facebook use (T1) and suicide-related outcomes (T2) was significantly mediated by RNT (T1). In contrast, a check test that included RNT (T1) as predictor and addictive Facebook use (T1) as mediator revealed no significant mediation effect. The current results demonstrate that if addictive Facebook use leads to RNT suicide ideation and attempts become likely. Therefore, RNT and addictive Facebook use should be taken into account when assessing individuals for risk of suicide.

## Introduction

Repetitive negative thinking (RNT) is defined as a style of perseverative thinking about one’s problems or negative experiences that is partly intrusive and difficult to disengage from (Ehring et al., 2011). The two most intensively studied types of RNT are worry and rumination: Worry has been defined as a predominantly verbal thought activity, which is negatively affect-laden, relatively uncontrollable, and focused on uncertain events with the potential for future negative outcome (Borkovec et al., 1988). In contrast, rumination refers to a type of repetitive thinking in response to sad mood. The individual dwells on the causes, meaning and implications of mood, as well as problems and events from the past (Nolen-Hoeksema, 1991). RNT (i.e., worry and rumination) has been identified as a vulnerability factor for the onset and maintenance of various mental disorders, such as depression, generalized anxiety disorder, social phobia, and posttraumatic stress disorder (Ehring & Watkins, 2008; Teismann & Ehring, 2019). The role of RNT in prolonging and intensifying depression and anxiety has been shown in various experimental, cross-sectional and longitudinal studies – using clinical and non-clinical samples of adults, adolescents, and children (e.g., Watkins, 2008). Moreover, experimental studies have provided evidence that RNT negatively biases thinking, impairs motivation, and interferes with problem-solving (Nolen-Hoeksema et al., 2008). Finally, RNT has been shown to mediate the association between a variety of other risk factors and depression (Spasojević & Alloy, 2001).

### Repetitive Negative Thinking and Suicide-Related Outcomes

In recent years, a close association between RNT, suicide ideation and suicide attempts has been shown in cross-sectional and longitudinal studies (Rogers & Joiner, 2017) – even when different types of RNT as well as different methodologies, samples (clinical and non-clinical) and measures of suicidality were used (Kerkhof & van Spijker, 2011; Morrison & O’Connor, 2008). For example, rumination was found to be more common in suicide attempters than in non-attempters (Krajniak et al., 2013) and uncontrollability of worry has been shown to be predictive of suicide ideation (Gorday et al., 2018). Furthermore, rumination significantly predicted suicide ideation in prospective studies using student and community samples (Krajniak et al., 2013; Miranda & Nolen-Hoeksema, 2007; Smith et al., 2006). Suicide-specific rumination outperformed a range of other suicide risk factors in predicting the presence of a lifetime suicide attempt (Rogers & Joiner, 2018). Taken together, there is strong empirical evidence on the importance of RNT for understanding suicide ideation and behavior. However, there is a lack of studies investigating the extent to which increased RNT mediates the association between other risk factors and suicide ideation and behavior.

### Associations of (Addictive) Facebook Use

Considering recent research results, use of online social media might belong to such risk factors. Studies that investigated large representative samples from the U.S. reported an increase of suicide ideation and behavior in young adults and adolescents in comparison to older generations. The enhanced tendency for excessive social media use – that is particularly high in younger generations – was assumed to be a potential reason for this development (Twenge et al., 2019a; Twenge et al., 2019b). With more than 1.8 billion daily users Facebook is currently the largest and the most popular social platform worldwide (Roth, 2021). Most earlier investigations that focused on the relationship between social media use and mental health were conducted on the social platform Facebook (Frost & Rickwood, 2017; Marino et al., 2018b; Ryan et al., 2014; Verduyn et al., 2017). Cross-sectional as well as longitudinal studies reported intensive Facebook use to be positively associated with loneliness, negative mood, depression, and anxiety symptoms (Kaye, 2019; Marino et al., 2018a; Skues et al., 2017; Tanhan et al., 2020). Furthermore, it was positively linked to less life satisfaction and less positive mood (Kross et al., 2013; Shakya & Christakis, 2017; Tromholt, 2016).

Moreover, intensive Facebook activity was assumed to contribute to the development of an emotional bond to the social platform that is associated with a strong problematic need to stay permanently online – a phenomenon that was termed as addictive Facebook use (Andreassen et al., 2012; Brailovskaia & Margraf, 2017). Addictive Facebook use was defined by six typical characteristics: salience (i.e., permanent thinking of Facebook use), tolerance (i.e., enhanced time has to be spent on Facebook to experience positive emotions), mood modification (i.e., Facebook is used for mood improvement), relapse (i.e., reverting to old use pattern despite endeavors to reduce Facebook activity), withdrawal symptoms (i.e., feeling nervous without Facebook use), and conflicts (i.e., interpersonal problems because of the high intensity of Facebook use) (Andreassen et al., 2012). Notably, addictive Facebook use has not been recognized as a formal psychiatric disorder in the diagnostic and statistical manual of mental disorders (DSM-5; American Psychiatric Association, 2013) or in the international classification of diseases (ICD-11; World Health Organization, 2018). Furthermore, some researchers emphasize that it is important not to over pathologize intensive online activity (Billieux et al., 2015; Carbonell & Panova, 2017).

Nevertheless, it is important to consider that cross-sectional studies reported addictive Facebook use to be positively related to the experience of daily stress, anxiety symptoms and sleep problems (Andreassen et al., 2012; Atroszko et al., 2018; Brailovskaia et al., 2019b; Brailovskaia et al., 2019c; Koc & Gulyagci, 2013). In a longitudinal study that investigated clinical patients, addictive Facebook use was positively associated with the level of depressive symptoms and insomnia up to six weeks later (Brailovskaia et al., 2019a). In a further longitudinal study that focused on university students, it was positively linked to suicide ideation and suicide behavior that were assessed one year later (Brailovskaia et al., 2020a). So far, the mechanisms that may explain these findings remain unclear. Against the presented empirical background, the following considerations might contribute to their explanation at least partly. Addictive Facebook use is linked to interpersonal problems in the offline world. The excessive use contributes to the neglect of one’s obligations and therefore often evokes conflicts at work and at home (Atroszko et al., 2018; Marino et al., 2018b). These negative experiences could foster RNT as a form of a dysfunctional coping-strategy and RNT could contribute to suicide-related outcomes (Krajniak et al., 2013). Thus, one may speculate that enhanced RNT might be a mechanism linking addictive Facebook use to suicide ideation and behavior.

### Study Aims and Hypotheses

Against this background, we aimed to investigate the relationship between addictive Facebook use, suicide-related outcomes and RNT within a longitudinal design – two measurement time points (T1 and T2) with a one-year time interval. We expected suicide-related outcomes (T2) to be positively linked to addictive Facebook use (T1) (Hypothesis 1a), as well as to RNT (T1) (Hypothesis 1b). RNT (T1) was assumed to positively mediate the association between addictive Facebook use (T1) and suicide-related outcomes (T2) (Hypothesis 2). The findings of our study should contribute to a better understanding of the mechanisms that could contribute to suicide-related outcomes.

## Methods and Materials

### Procedure and Participants

The present study has a longitudinal design. We assessed data in October 2018 (= T1) and in October 2019 (= T2). At T1, 250 individuals who study/have studied at a large German university in the Ruhr region were contacted by e-mail that included a participation invitation and a link for the first online survey. All of them had previously expressed willingness to be contacted for research investigations. Participation was voluntary and not compensated. The requirement for participation was a current Facebook membership. At T2, the 204 participants who completed the first survey were contacted again by e-mail to complete the second online survey. Both surveys were completed by 191 persons (73.8% women; T1: *M*_*age*_ = 26.17, *SD*_*age*_ = 6.63, range:18–55; occupation: 59.7% students, 40.3% employees; T2: 52.9% students, 47.1% employees). Of the 191 participants, 2.1% (*n* = 4) reported lifetime suicide attempts at T2. Independent t-tests revealed no significant group differences between attempers and non-attempers considering demographic variables, RNT, and addictive Facebook use. Therefore, all statistical analyses were conducted with the overall sample. The implementation of the present study was approved by the responsible Ethics Committee. All participants were properly instructed and provided their informed consent online. A priori conducted power analyses (G*Power program, version 3.1) showed that the sample size was sufficient for valid results (power > .80, *α* = .05, effect size *f*^*2*^ = 0.15; cf., Mayr et al., 2007).

### Measures

#### Addictive Facebook Use

The brief version of the Bergen Facebook Addiction Scale (BFAS) (Andreassen et al., 2012) measured the level of addictive Facebook use with six items (e.g., “Felt an urge to use Facebook more and more?”) that correspond to the six characteristics of addictive Facebook use (salience, tolerance, mood modification, relapse, withdrawal, conflict). Items were rated on a 5-point Likert-type scale (1 = *very rarely*, 5 = *very often*; current internal consistency: Cronbach’s *α* = .66). Higher sum scores indicate higher levels of addictive Facebook use.

#### Repetitive Negative Thinking (RNT)

The level of RNT was assessed with two items that were construed by the authors based on available longer RNT measures (see Perseverative Thinking Questionnaire, PTQ; Ehring et al., 2011). The items focused, respectively, on one of the two RNT forms that are worry (“I am often worried”) and rumination (“I often tend to ruminate”). They were rated on a 5-point Likert-type scale (1 = *does not apply to me at all*, 5 = *applies to me very much;* current internal consistency: *α* = .83). Higher sum scores indicate higher levels of RNT. Notably, available research described the validity, reliability, and efficacy of single-item measures especially in online surveys and encouraged their use (Konrath et al., 2014; Szrek et al., 2012).

#### Suicide-Related Outcomes

To assess suicide-related outcomes Item 1 (“Have you ever thought about or attempted to kill yourself?”) of the Suicidal Behaviors Questionnaire-Revised (SBQ-R) (Osman et al., 2001) was rated on a 6-point Likert-type scale (1 = *never*, 6 = *I have attempted to kill myself, and really hoped to die*). Previous research revealed this item to be a valid instrument for brief screening purposes of suicide-related outcomes. It has been repeatedly used in clinical and non-clinical samples (Green et al., 2015; Osman et al., 2001).

### Statistical Analyses

Statistical analyses were conducted with SPSS 26 and the macro Process version 3.5 (www.processmacro.org/index.html) (see Hayes, 2013). First, descriptive statistics and zero-order bivariate correlations between the investigated variables were calculated. Next, a mediation analysis (model 4) was investigated that included addictive Facebook use (T1, predictor), RNT (T1, mediator), and suicide-related outcomes (T2, outcome); considering the mostly young and female composition of the present sample, age and gender (both T1) were controlled for by including both as covariates. The basic association between addictive Facebook use (T1) and suicide-related outcomes (T2) was denoted by *c* (the total effect). The path of addictive Facebook use (T1) to RNT (T1) was denoted by *a*, and the path of RNT (T1) to suicide-related outcomes (T2) was denoted by *b*. The combined effect of path *a* and path *b* presented the indirect effect. The direct effect of addictive Facebook use (T1) to suicide-related outcomes (T2) after inclusion of RNT (T1) in the model was denoted by *c’*. The mediation effect was assessed by the bootstrapping procedure (10.000 samples) that provides percentile bootstrap confidence intervals (95%CI). Considering that both addictive Facebook use and RNT were assessed at T1, a further mediation analysis was undertaken that included RNT as a predictor, addictive Facebook use as mediator, and suicide-related outcomes as outcome as a check test.

## Results

Table 1 presents the descriptive statistics of the investigated variables as well as their correlations. Addictive Facebook use (T1) was significantly positively correlated with RNT (T1) and suicide-related outcomes (T2). RNT (T1) and suicide-related outcomes (T2) were also significantly positively correlated (see Table 1).

**Table 1:** Descriptive statistics and correlations of the investigated variables

	*M (SD)*	*Min–Max*	(2)	(3)
(1) Addictive Facebook Use (T1)	7.82 (2.41)	6–16	.150*	.171*
(2) Repetitive Negative Thinking (T1)	6.56 (2.14)	2–10		.253**
(3) Suicide-Related Outcomes (T2)	2.00 (1.05)	1–6		

*N* = 191; *M* = Mean; *SD* = Standard Deviation; *Min* = Minimum; *Max* = Maximum; **p < .01; *p < .05

**Fig. 1:**
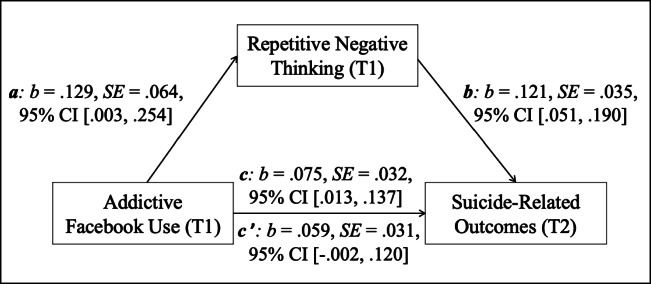
Mediation model with addictive Facebook use (T1, predictor), repetitive negative thinking (T1, mediator), and suicide-related outcomes (T2, outcome). *Note. N* = 191; *c* = total effect, *c’* = direct effect; *b* = standardized regression coefficient, SE = standard error, CI = confidence interval

Results of the bootstrapped mediation analysis revealed that RNT (T1) significantly mediated the relationship between addictive Facebook use (T1) and suicide-related outcomes (T2) (see Fig. 1). The basic association between addictive Facebook use (T1) and suicide-related outcomes (T2) was significant (total effect, *c*: *p* = .019). After the inclusion of RNT (T1) in the model, the link between both variables was no longer significant (direct effect, *c’*: *p* = .058). The relationships between addictive Facebook use (T1) and RNT (T1) (*a*: *p* = .045), and the link between RNT (T1) and suicide-related outcomes (T2) (*b*: *p* = .001) were significant, as well as the indirect effect (*ab*), *b* = .016, SE = .009, 95% CI [.001, .037].

The check test revealed that addictive Facebook use (T1) did not mediate the association between RNT (T1) and suicide-related outcomes (T2). The total effect was significant (*c*: *b* = .130, SE = .035, 95% CI [.061, .200], *p* < .001). After the inclusion of the mediator in the model, the direct effect was also significant (*c’*: *b* = .121, SE = .035, 95% CI [.051, .190], *p* < .001) and comparably high as the total effect. The indirect effect (*ab*) was not significant (*b* = .010, 95% CI [−.002, .027].

## Discussion

In the present longitudinal study, RNT and addictive Facebook use were found to be positively associated with suicide-related outcomes one year later (confirmation of Hypothesis 1a and Hypothesis 1b). Moreover, RNT mediated the association between addictive Facebook use and suicide-related outcomes (confirmation of Hypothesis 2). A check test revealed that addictive Facebook use did not mediate the relationship between RNT and suicide-related outcomes (further confirmation of Hypothesis 2).

The present findings complement previous studies showing that RNT can predict suicide ideation and suicide behavior up to one year later (Rogers & Joiner, 2017). Ruminative thinking is associated with a negatively biased thinking style (Spasojević & Alloy, 2001), hopelessness (Smith et al., 2006), perceptions of entrapment (Teismann & Forkmann, 2017), as well as overarousal (Rogers et al., 2017). These effects might contribute to a rapid mutual build-up of RNT and increasing negative mood leading to a state of aversive negative emotionality associated with suicide ideation/behavior. The current results underline the importance of RNT for understanding suicide-related outcomes. They also complement previous findings showing that RNT mediates the relationship between different risk factors and psychopathological outcomes (Spasojević & Alloy, 2001).

Furthermore, the current study replicates – within an independent sample – recent findings that social media use (Twenge et al., 2018), specifically addictive Facebook use (Brailovskaia et al., 2020a), is prospectively associated with suicide-related outcomes. Moreover, they contribute to a better understanding of mechanisms that may mediate the relationship between addictive Facebook use and suicide-related outcome. It seems that addictive tendencies of Facebook use might contribute to RNT. Individuals with enhanced levels of addictive Facebook use often experience interpersonal conflicts (Kaye, 2019). At worst, these conflicts may result in loss of important social relationships. This negative experience might contribute to feelings of failure and decrease of self-esteem that positively predict RNT (Kernis et al., 1991). The link between addictive Facebook use and RNT might also be enhanced by experiences of relapse. Notably, individuals with increased levels of addictive Facebook use who try to reduce their Facebook activity on their own often are not able to handle this challenge (Andreassen & Pallesen, 2014). This might enhance further feelings of failure. In the longer-term, RNT could foster suicide ideation and suicide behavior (Rogers & Joiner, 2017).

### Implications

The current findings are of specific interest considering the global outbreak of the coronavirus disease (Covid-19; severe acute respiratory syndrome coronavirus 2, SARS-CoV-2) in the year 2020 (World Health Organization, 2020), and the recent increase of suicide-related outcomes (Tanaka & Okamoto, 2021). To fight the pandemic spread, many governments introduced restrictive rules such as the need to maintain distance from other people and to limit offline meetings (“social distance”) (Tso & Cowling, 2020). As a consequence, social platforms such as Facebook became one of the main sources of social interaction. Their use significantly increased (Cellini et al., 2020; Tanhan et al., 2020). Also the tendencies of addictive online behavior increased (Masaeli & Farhadi, 2021). Notably, the data of the present study were collected in the years 2018 and 2019 before the Covid-19 outbreak. They reveal potential mechanisms that underly the link between addictive online behavior and suicide-related outcomes. Therefore, we strongly recommend future research to investigate our mediation model with a more recently collected data set. Its replication could at least partly explain the enhancement of suicide-related outcomes during the Covid-19 outbreak. Moreover, it would emphasize the need for governmental prevention programs that reduce online time and focus on training of competent social media use. Such programs could for example be implemented in schools and other educational institutions.

Clinically, the present data suggest that it could be useful to incorporate RNT and addictive Facebook use – together with established predictors – into the psychosocial risk assessment of persons contemplating suicide. Furthermore, addressing RNT could be a viable target for psychotherapeutic interventions in the prevention of suicide. Previous research described cognitive behavioral based group psychotherapy (CBGP) and brief cognitive behavioral therapy (CBT) focusing on the reduction of RNT (by the inclusion of rumination and worry management as well as mindfulness) to have a significant positive effect on mental health (e.g., reduction of depression and anxiety) (Baeken et al., 2021; Kertz et al., 2015). Furthermore, Williams et al. (2017) advocated mindfulness-based cognitive therapy (MBCT) in preventing recurrent suicidal ideation and behavior. By training the ability to maintain the focus of attention on the present moment, mindfulness is proposed to inhibit dysfunctional repetitive thought processes and to predict positive effects on mental health (Tanhan, 2020; Williams et al., 2017). In line with this assumption, training in mindfulness has been shown to reduce ruminative thinking (Perestelo-Perez et al., 2017), as well as suicide ideation (Forkmann et al., 2016).

MBCT could also reduce tendencies of addictive Facebook use, as mindfulness (i.e., the enhanced attention to and the nonjudgmental awareness of the present moment; Bishop et al., 2004) was described to be negatively linked to excessive use of social media (Apaolaza et al., 2019). Individuals with enhanced levels of addictive Facebook use often consider Facebook use as a possibility to escape from daily problems and obligations. During the immersion into the online world they lose the sense of time and experience difficulties in maintaining the focus of attention on happenings in the offline world (Brailovskaia et al., 2018a). Mindfulness could prevent the negative consequences.

Furthermore, the Acceptance and Commitment Therapy (ACT) (Hayes et al., 2012; Tanhan, 2019), specifically the Repetitive Negative Thinking-focused ACT (RNT-focused ACT) (Ruiz et al., 2016) could also serve as a protective strategy against the interaction between addictive Facebook use and RNT and its impact on suicide-related outcomes. Identification of main triggers of RNT, taking distance from them, and the focus on valued actions that are important for the individual in the longer-term belong to the different training steps of RNT-focused ACT (Ruiz et al., 2020). These steps could support the individual to identify the potential negative effect of excessive online behavior and to determine valued alternative behavior. Following earlier studies (Brailovskaia et al., 2018b; Klaperski et al., 2013), such behavior might be sportive activity (for example jogging or cycling). It can reduce the risk of addictive Facebook use and improve mental health (Brailovskaia & Margraf, 2020; Harris et al., 2006; Southerland et al., 2016). Individuals concerned could be advised to involve sportive activity in their daily life. Engagement in sportive activity and the achievement of small self-determined sportive aims (such as the increase of own running speed) can enhance one’s self-esteem and foster positive emotions (Brailovskaia et al., 2020b; Wunsch et al., 2017). This can reduce the need to search for positive emotions in the online world as well as the trigger function of addictive Facebook use for RNT, and thus the tendency for RNT. Especially adolescents and young adults who tend to intensive online activity and show decreased levels of mental health (Twenge et al., 2018) might benefit from the involvement of sportive activity in their everyday life as an alternative to Facebook use.

### Limitations

The current study has some limitations that should be considered. First, the sample included mostly young female participants, which limits the generalizability of the present results. Second, suicide-related outcomes were measured with only one item. Even though this approach is common for screening purposes (Osman et al., 2001) and there is strong evidence for the predictive ability and relevance of single items assessing suicide ideation (Green et al., 2015), future studies using multidimensional instruments that consider suicidal ideation and suicidal behavior separately are recommended. The same is true for the measure of RNT that included only two self-developed items. At the same time, it is interesting that in the present study an association between RNT and suicide-related outcomes could be shown, although only a comparatively rudimentary measure was used to assess RNT. Moreover, even though the BFAS is a well-established instrument to assess addictive Facebook use (Andreassen et al., 2012), in the present sample its internal consistency was low. This should be considered when interpreting the present results. Third, suicide-related outcomes were assessed only at the second measurement time point. Therefore, no conclusions about their potential changes in relationship with addictive Facebook use and RNT between T1 and T2 can be drawn. Fourth, in the present study, the focus was on addictive use of Facebook. Considering that recent research described potential negative impact of excessive use of other social platforms such as Twitter and Instagram on mental health (Longobardi et al., 2020; Rozgonjuk et al., 2020), future studies are suggested to replicate the present findings for addictive social media use in general. They could investigate whether the potential negative effect of addictive social media use in general and its interaction with RNT can foster suicide-related outcomes, or whether the mediation model is specific for addictive Facebook use. Fifth, in the present study, the main focus was only on RNT as a potential mediator between addictive Facebook use and suicide-related outcomes. Considering that both addictive Facebook and suicide-related outcomes are complex constructs that can be linked and influenced by different factors (Marino et al., 2018b; Rogers & Joiner, 2018), future research should extend the current meditation model by further potential confounding variables such as personality traits, impulsivity, stress, and anxiety symptoms. Moreover, earlier research (e.g., Tanhan & Strack, 2020) emphasized the need to focus not only on intrapersonal factors but also on environmental conditions as suggested by the Ecological Systems Theory (EST) (Bronfenbrenner, 1977) to understand individual behavior and mental health. Therefore, further potential confounding variables such as the family background, societal and cultural factors should be included. Sixth, future studies might complement the quantitative data assessment via online surveys that we used in the present study by further more comprehensive qualitative methods. One of such methods might be the Online Photovoice (OPV) that can provide deeper insight into the individual perception and reaction to different environmental factors than online surveys only (see Tanhan & Strack, 2020).

To conclude, the current study highlights the importance of perceptions of RNT in understanding suicide ideation and suicide behavior. Furthermore, the present results point to the fact that the assessment of social media use habits, especially tendencies of addictive Facebook use, could support the identification of individuals at risk for suicide-related outcomes. Moreover, they allow the assumption – which should be further investigated – that an early identification of addictive Facebook use and RNT tendencies, and their thematization in the frame of the therapeutic setting might reduce one’s risk for suicide-related outcomes. This might be of specific importance since the outbreak of Covid-19 and the enhanced use of Facebook and other social platforms.

## Data Availability

The dataset and further material analysed during the current study will be available from the corresponding author on reasonable request.
